# Development of the Centrifugal Blood Pump for a Hybrid Continuous Flow Pediatric Total Artificial Heart: Model, Make, Measure

**DOI:** 10.3389/fcvm.2022.886874

**Published:** 2022-08-04

**Authors:** Carson S. Fox, Thomas Palazzolo, Matthew Hirschhorn, Randy M. Stevens, Joseph Rossano, Steven W. Day, Vakhtang Tchantchaleishvili, Amy L. Throckmorton

**Affiliations:** ^1^School of Biomedical Engineering, Science and Health Systems, Drexel University, Philadelphia, PA, United States; ^2^St. Christopher's Hospital for Children, Philadelphia, PA, United States; ^3^Division of Cardiology, Children's Hospital of Philadelphia, Philadelphia, PA, United States; ^4^Department of Biomedical Engineering, Kate Gleason College of Engineering, Rochester Institute of Technology, Rochester, NY, United States; ^5^Cardiac Surgery, Thomas Jefferson University, Philadelphia, PA, United States

**Keywords:** pediatric mechanical circulatory support, pediatric total artificial heart, rotary blood pump, rotary blood pump modeling, pediatric ventricle assist device, pediatric ventricular support

## Abstract

Clinically-available blood pumps and total artificial hearts for pediatric patients continue to lag well behind those developed for adults. We are developing a hybrid, continuous-flow, magnetically levitated, pediatric total artificial heart (TAH). The hybrid TAH design integrates both an axial and centrifugal blood pump within a single, compact housing. The centrifugal pump rotates around the separate axial pump domain, and both impellers rotate around a common central axis. Here, we concentrate our development effort on the centrifugal blood pump by performing computational fluid dynamics (CFD) analysis of the blood flow through the pump. We also conducted transient CFD analyses (quasi-steady and transient rotational sliding interfaces) to assess the pump's dynamic performance conditions. Through modeling, we estimated the pressure generation, scalar stress levels, and fluid forces exerted on the magnetically levitated impellers. To further the development of the centrifugal pump, we also built magnetically-supported prototypes and tested these in an *in vitro* hydraulic flow loop and *via* 4-h blood bag hemolytic studies (*n* = 6) using bovine blood. The magnetically levitated centrifugal prototype delivered 0–6.75 L/min at 0–182 mmHg for 2,750–4,250 RPM. Computations predicted lower pressure-flow performance results than measured by testing; axial and radial fluid forces were found to be <3 N, and mechanical power usage was predicted to be <5 Watts. Blood damage indices (power law weighted exposure time and scalar stress) were <2%. All data trends followed expectations for the centrifugal pump design. Six peaks in the pressure rise were observed in the quasi-steady and transient simulations, correlating to the blade passage frequency of the 6-bladed impeller. The average N.I.H value (*n* = 6) was determined to be 0.09 ± 0.02 g/100 L, which is higher than desired and must be addressed through design improvement. These data serve as a strong foundation to build upon in the next development phase, whereby we will integrate the axial flow pump component.

## Clinical Significance and Motivation

Globally, more than 17.5 million patients die of cardiovascular disease, the leading cause of death worldwide (1). Congestive heart failure (CHF), which is a result of end-stage cardiovascular disease, affects more than 60 million patients around the world (2). The clinical need is expected to increase, and the necessity for effective therapeutic strategies is of critical importance, especially for infants and children. Millions of pediatric patients around the world die each year from heart failure due to congenital or acquired cardiac diseases (3). Complex congenital heart defects and exposure to viruses and bacteria that attack the cardiac muscle lead to severely depressed ventricular function and usually require surgical and therapeutic intervention (4, 5). Annually, more than 10,000 children in the United States are hospitalized with CHF-related symptoms, with a mortality rate of 7–15% (6–8). These pediatric patients often require a heart transplantation, the current standard of care. Transplantation in pediatric patients is complicated by challenges in donor-recipient size matching and anticipated growth potential (9). Despite ~74% of children being matched and transplanted within 90 days of listing, the mortality rate range for patients awaiting a donor organ remains high (5–39%) (7, 10, 11). Thus, this necessitates the use of alternative therapies, such as mechanical circulatory support (MCS) devices, including blood pumps.

MCS devices, such as ventricular assist devices (VADs) and total artificial hearts (TAHs) have been employed to support pediatric patients with some success (12). The Berlin Heart EXCOR (Berlin Heart, Berlin Germany) has been employed for thousands of pediatric patients, and Abbott Laboratories received U.S. FDA labeling for use of the HeartMate III in older pediatric patients with advanced refractory left ventricular HF in December 2020. On a case-by-case basis, blood pumps, such as the Jarvik Heart (Jarvik Heart Inc., New York, NY) and HeartMate II axial flow pump, may be employed for pediatric compassionate use. The SynCardia TAH has 2 sizes (50 and 70 cc) that both have limitations of use in smaller pediatric patients. Researchers at the Cleveland Clinic have multiple TAHs under development, including a standard sized device for adults and adolescents, and a pediatric device, which is 1/3 the total volume of the adult device.

**Figure 1:**
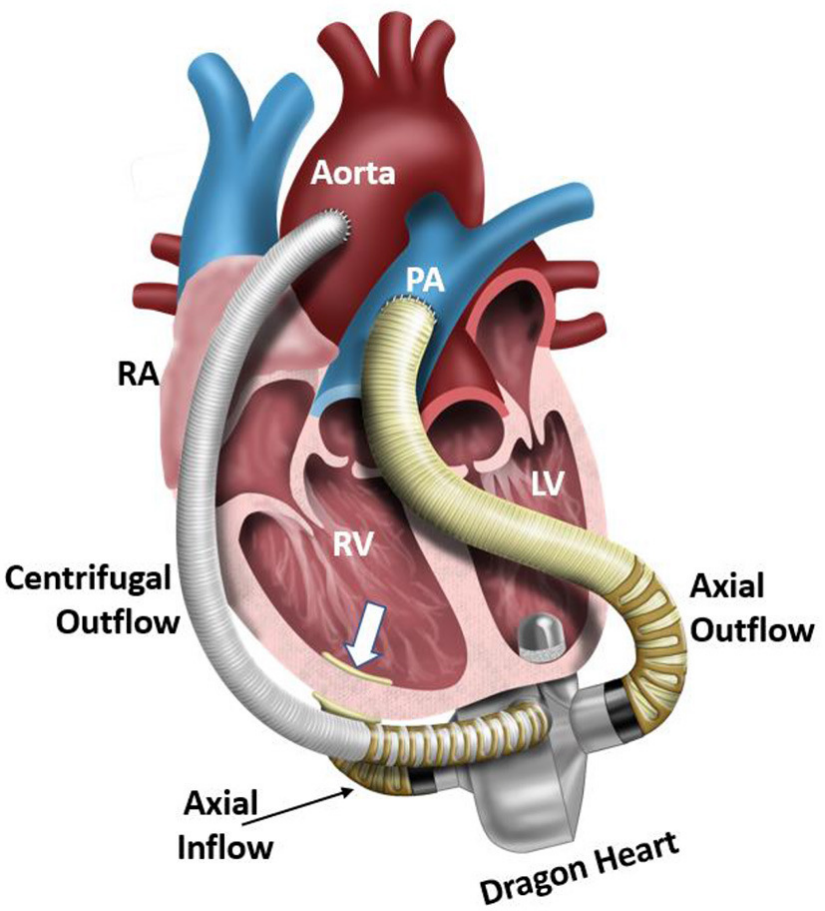
Illustration of an implanted Dragon Heart hybrid continuous-flow blood pump technology. The centrifugal blood pump is designed to support the systemic circulation and left ventricle, and the axial flow blood pump is designed to support the pulmonary circulation and right ventricle. In this study, we focus on the design-build-test of the centrifugal blood pump component of this device. RA, right atrium; RV, right ventricle; LV, left ventricle; PA, pulmonary artery.

**Figure 2:**
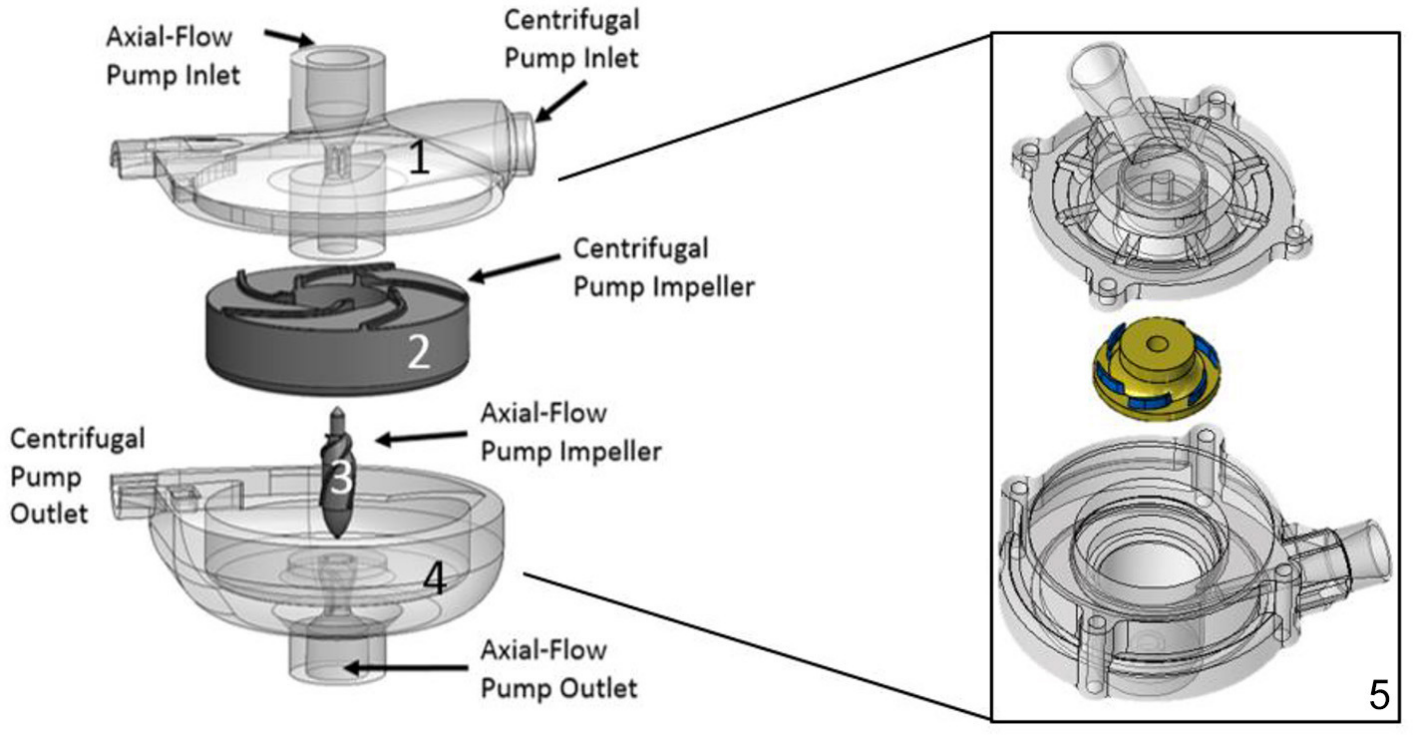
Hybrid continuous-flow TAH. (1) Upper housing, (2) Centrifugal pump impeller, (3) Axial pump impeller, (4) Lower housing. This configuration consists of two separate pump domains combined into one pump housing, (5) Concentrated design focus on the centrifugal pump design only: Reduced overall size with an improved and compact centrifugal impeller and pump design, overall 60 mm in diameter by 50 mm in height, having a radial inlet volute and 37 mm impeller diameter (6 bladed design).

Development and translation of pediatric MCS devices continue to lag well behind those developed for adults. To address the ongoing limitations of existing MCS devices for pediatric patients, we are developing a hybrid, continuous-flow, implantable or extracorporeal, magnetically levitated, TAH for pediatric patients (13, 14). This new device has the capability to be employed as a right-sided or left-sided VAD, or TAH for partial or full pediatric cardiovascular support. The hybrid TAH design integrates both an axial and centrifugal blood pump within a single, compact housing. Figure 1 displays an illustration of the clinical implantation of the devices during pediatric MCS of both the left ventricle (centrifugal blood pump) and the right ventricle (axial blood pump). The centrifugal pump rotates around the separate axial pump domain, and both impellers rotate around a common central axis. In this configuration, the axial flow blood pump serves to provide MCS to the pulmonary circulation, and the centrifugal flow blood pump supports blood flow in the systemic circulation. The internal axial flow pump is designed to generate 1–6 *L/min* of flow with a pressure rise range of 10–30 *mmHg* for the pulmonary circulation, and the outer centrifugal blood pump is designed to produce 60–140 *mmHg* for the systemic circulation at rotational speeds to 10,000 RPM. This range of pressure generation and flow capacity is required to support a pediatric cardiovascular load. This constitutes the first-ever technology to integrate an axial and centrifugal blood pump as a TAH. Figure 2 illustrates this design concept, also referred to as the *Drexel Dragon Heart*. Since the blood pump domains are separate, we have to-date concentrated on development of the centrifugal blood pump, which will contribute most substantially to the overall size of the device. The centrifugal flow pump has an inlet volute, exit volute, and magnetically levitated impeller. The inlet volute supports flow entering the pump and distributes the flow evenly into the impeller region. The exit volute directs blood flow out of the pump by slowly increasing the cross-sectional area to convert fluid velocity to pressure. The development results presented in this manuscript reflect the latest design work for the centrifugal blood pump, after 4 design phases.

Here, we performed computational fluid dynamics (CFD) analysis of the blood flow through the centrifugal pump during steady and transient conditions. To initiate design of the motor and magnetic suspension system, we conducted a transient CFD analysis on the magnetically suspended centrifugal blood pump design to assess its dynamic performance conditions. The suspension system must compensate for both fluid disturbances and force perturbations on the levitated rotor-impeller to consistently maintain its optimally centered position during operation. Transient simulations are utilized to investigate more dynamic estimations of fluid disturbances and to quantify the fluid forces that are required to the design and development the robust magnetic suspension and motor (15–17). Three types of simulations were investigated: (1) steady flow simulations using a frozen-rotor interface specification; (2) quasi-steady simulations whereby the impeller is rotated manually and a steady state simulation is performed; (3) transient rotational sliding interfaces (TRSI), where the rotor-stator domains of the impeller move in relative motion to each other, simulating blade rotation. Through computational modeling, we estimated the pressure generation, scalar stress levels, and fluid forces exerted on the magnetically levitated impeller. To further the development of the centrifugal pump, we also built prototypes of the designs and tested those in an *in vitro* hydraulic flow loop and by blood bag hemolytic studies. The findings of these analyses are described in subsequent sections.

## Materials and Methods

### Computational Studies

**Figure 3:**
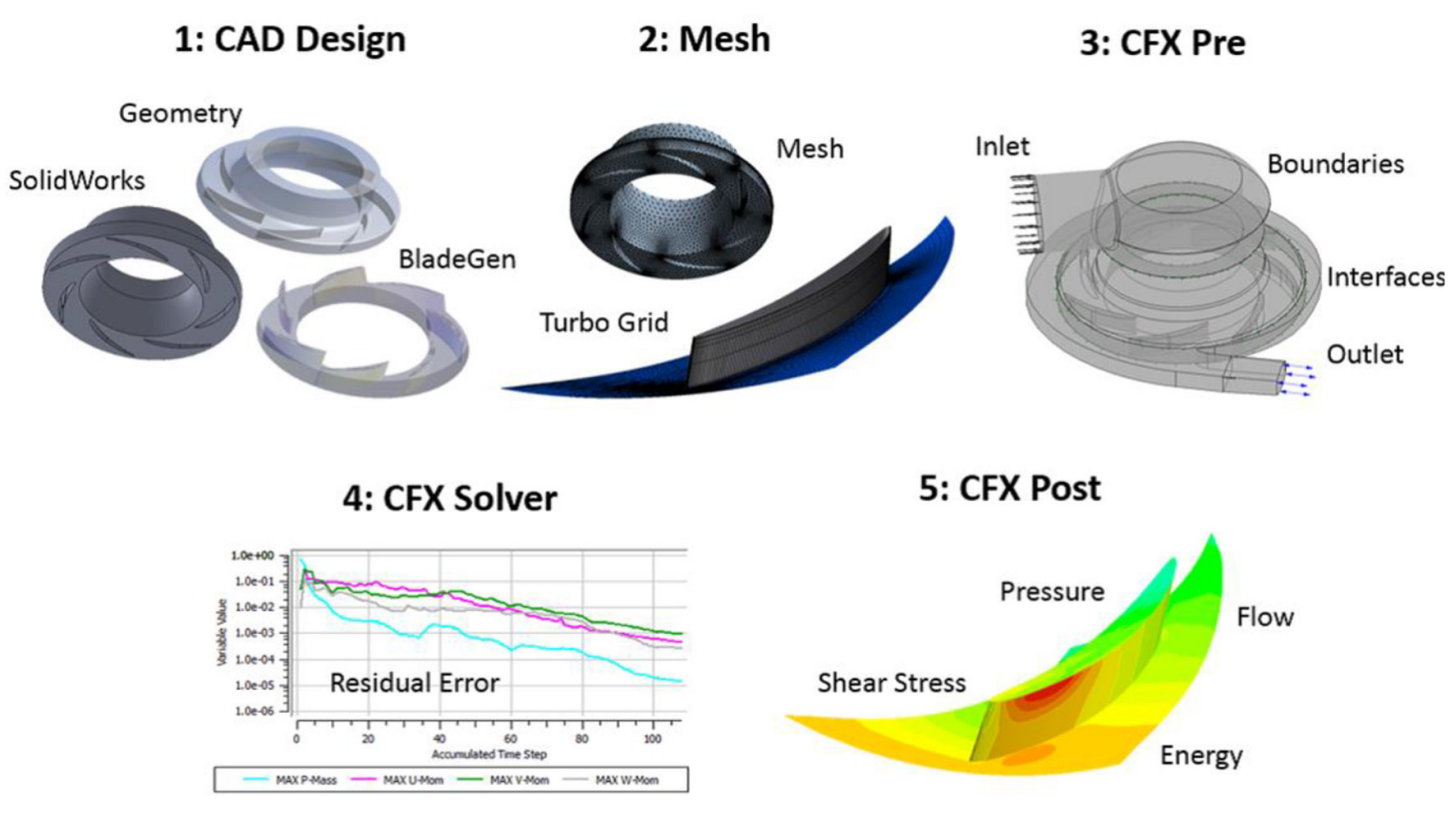
Computational modeling approach using ANSYS CFX. (1) SolidWorks CAD design of the pump, (2) ANSYS meshing of pump regions, (3) ANSYS CFX-Pre to define physics and boundary conditions, (4) ANSYS CFX solver to complete the simulation, and (5) ANSYS CFX-post to quantify and visualize the pump performance metrics (pressure generation, fluid stress levels, velocity profiles). For CFX-post, nodal properties are exported to a Matlab code in order to estimate the blood damage index for a given operating condition. As an exemplar of the approach, a contour plot of a single bladed centrifugal impeller is illustrated.

**Figure 4:**
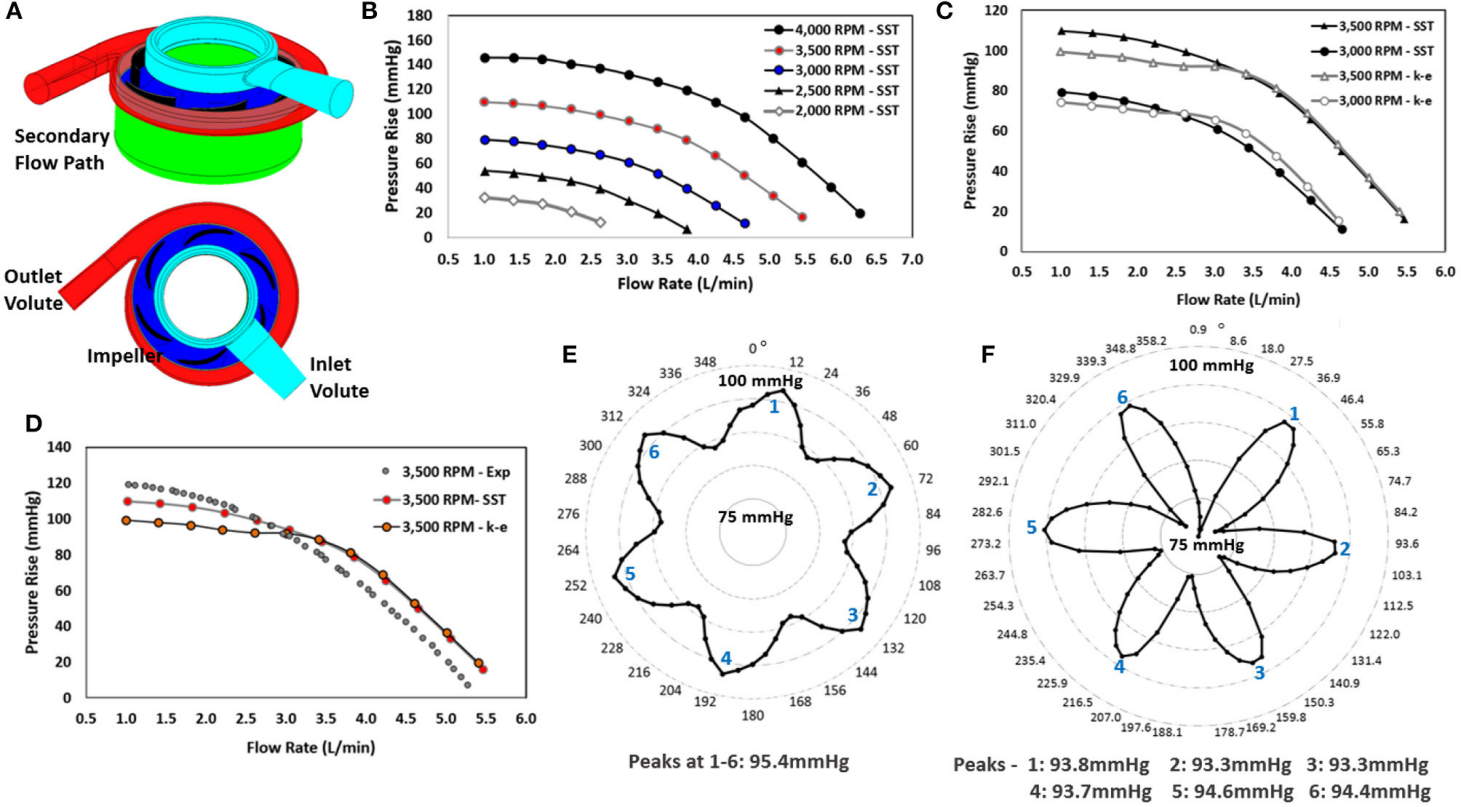
Computational modeling: **(A)** Model with regions labeled, **(B)** Pressure-flow performance curves for operating conditions of 3,000 to 4,000 RPM at flow rates of 1 to 6 *L/min* for the SST turbulence model, **(C)** Comparison of pressure-flow performance curves for the SST and k-epsilon turbulence models for operating conditions of 3,000 to 4,000 RPM at flow rates of 1 to 5 *L/min*, **(D)** Qualitative comparison of experimental hydraulic data for the 3,500 RPM operating speed with the computational predictions of the k-epsilon and SST turbulence models, **(E)** Pressure rise across the centrifugal pump using a quasi-steady analysis, **(F)** Pressure rise across the centrifugal pump using TRSI analysis.

We employed ANSYS-CFX (ANSYS Inc., Canonsburg, PA) to simulate both steady and transient blood flow through the centrifugal pump, and the approach is shown in Figure 3. The centrifugal pump model (Figure 4A) consisted of several fluid domains: (1) inlet volute; (2) impeller; (3) outlet volute; and (4) the secondary flow path. Each of these domains were connected *via* fluid-fluid interfaces. We determined the global Reynolds number using the standard formula for pumps: ρωD^2^/μ, where ω is the angular speed, D signifies impeller diameter, and μ is fluid viscosity. Global and local Reynolds numbers were calculated to above 4,000, leading us to implement a turbulence model. Both the k-epsilon turbulence and shear stress transport (SST) model were employed. The shear stress transport (SST) turbulence model was selected because it combines the robustness of the k-ε in the bulk flow regime and the k-ω model along the surfaces or boundaries. A grid independence study was performed to determine when the grid resolution was no longer affecting the physics being modeled; we examined parameter values of velocity (radial and axial) and pressures at more than 10 locations in the centrifugal pump. Mesh quality was confirmed using standard mesh metrics, including aspect ratio, Jacobian ratio, skewness and an ANSYS metric called element quality. All surfaces were set to a no-slip condition and the simulations were executed using a high resolution advection scheme. The convergence criterion was set to a maximum residual threshold below 1 × 10^−3^.

#### Fluid Physics

Blood is well-known to be a Non-Newtonian fluid (18); it has been established, however, that a Newtonian fluid assumption is valid above a threshold strain rate whereby the stress-strain relationship of blood is primarily linear, indicating Newtonian behavior (18, 19). For k-epsilon turbulence modeling, we employed a Newtonian behavior model with a constant viscosity of 0.0035 Pa^*^s, corresponding to patient hematocrit of ~33% (20). In contrast, for the SST turbulence studies, we implemented a Non-Newtonian model based on a Generalized Oldroyd-B (GOB) model using relevant coefficients found in literature (18, 19). This approach utilizes a form of the Naiver-Stokes where the stress tensor is separated in terms of blood plasma components (**τ_s_**) and red blood cell components (**τ_p_**), as shown in Equation (1):


(1) $$\begin{array}{r} \rho \frac{\partial \vec{u}}{\partial t}+\rho \vec{u}\cdot \nabla \vec{u}=-\nabla p+\nabla \cdot \left(\tau_{s}+\tau_{p}\right) \end{array}$$

where **ρ** signifies the fluid density, **p** represents the pressure, and $\vec{u}$ is the fluid velocity vector.

The red blood cell stress tensor component includes a dissipative term using the Oldroyd derivative (τ'_p_), which relates the red blood cells stresses to the rate of change of the stress. In the model developed by *Good* et al. (18), a parameter limited term with constant α = 0.5 was included to mathematically bound the predicted stress. The coefficients of this model were experimentally derived using pediatric blood, including two coefficients (η, η_1_) bounding the viscosities as the shear rates approach zero and infinity. The elastic shear modulus (μ) was determined by finding a least squares fit of experimental data. In this study, we utilized this model and selected the closest hematocrit option of 40% and therefore the η, η_1_, and μ were defined as 31.1, 8.9, and 53, respectively (19). We specified a fluid density of 1,050 kg/m^3^ for these simulations.

#### Modeling Reference Frames

Two reference frames (rotating and stationary domains) were established in these simulations. The impeller domain was specified to be in the rotating reference frame. The inlet volute, outlet volute and secondary flow path were assigned to the stationary reference frame. ANSYS CFX provides two main options to define the interfaces between two differing frames of reference: frozen rotor and rotor/stator interfaces. In the frozen rotor mode, the two frames of reference link with a fixed relative position, but have the correct frame transformation occurring across the interface. This frame transformation occurs *via* a patch mapping technique to interpolate mass flux across interfaces of varying reference frames (i.e., Frozen Rotor Interface). A transient rotor/stator simulation, however, involves two or more domains in relative motion to each other, which more accurately reflects the physics of a rotating impeller. The transient rotor/stator interface solution is updated with each time step as the relative position of the two-grid regions change with time. The frozen rotor (steady flow) simulation results were applied as the initial guess for the transient rotor/stator simulations. The transient interface treatment accounts for all interaction effects between components in relative motion to each other, but requires more calculation resources and time (15–17).

#### Boundary Conditions

##### Non-uniform Inflow Velocity

To simulate fully developed inlet flow, a non-uniform inflow velocity profile was implemented as the inlet boundary condition to the centrifugal blood pump. Equation (2) details the expression that was defined for the inlet velocity profile with a power variable (β) equal to 1/7 for turbulent inflow conditions. The radius of inflow boundary of the inlet volute is specified as R_max_ (4 mm), and a maximum fluid velocity (u_max_) is defined to achieve the target flow rates of ~1–5 L/min (0.25–1.55 m/s).


(2) $$\begin{array}{r} u\left(r\right)=u_{\max}{\left(1-\frac{r}{R_{\max}}\right)}^{\beta } \end{array}$$

##### Frozen Rotor Steady Flow Simulations

Flow through the centrifugal pump was initially modeled as steady state. The impeller rotor was specified as rotating in the counterclockwise direction in accordance with the blade orientation, and the impeller hub, in the secondary flow path domain, was specified as a rotating surface. The frozen rotor interface linked regions of differing reference frames. The non-uniform mass inflow rate and operational rotational speed were specified for each steady-flow simulation. The outflow pressure was set to be a constant, static pressure afterload (200 mmHg). Both the k-epsilon turbulence and the shear stress transport (SST) model were specified, and rotational speeds of 3,000, 3,500, and 4,000 RPM were modeled for flow rates of 1-6 L/min, set by establishing the maximum velocity value, *u*_*max*_ (0.25–1.55 m/s), in Equation (2).

##### Quasi-Steady Flow Simulations

The purpose of the quasi-steady study was to assess the impact of centrifugal impeller blade rotational position on pump performance and fluid dynamics. We incrementally rotated the impeller blades by 6° and created a new, separate model for each rotational position (i.e., 60 models at 6° impeller rotations to achieve a 360° full rotation). The inlet boundary condition was specified as a non-uniform velocity profile (u_max_ of 0.75 m/s; β of 1/7), and the outlet boundary condition was defined as a constant, static pressure afterload (200 mmHg). We ran 60 simulations to derive the full 360° time-averaged performance profile for the centrifugal pump at 3,500 RPM (15–17).

##### Transient Rotational Sliding Interfaces (TRSI) Simulations

The TRSI simulations involved the application of a non-uniform inflow velocity profile (u_max_ of 0.75 m/s; β of 1/7) and a rotational speed of 3,500 RPM according to the pump design point. The outflow boundary condition or aortic mean pressure (AOP) was set to a constant physiologic value. We employed the rotor-stator interface, rather than the frozen-rotor, for the regions of differing reference frames. The rotor-stator interface more accurately models the motion of the rotating impeller relative to the stationary pump internal housing. Since the rotational speed is relatively high in each case, this simulation requires a small time step to resolve the transient fluid dynamics and capture the impact of the blade passage frequency (BPF). Rotational increments of 4.725°, corresponding to a time step of 2.225 × 10^−4^ s, were implemented for 3,500 RPM. This set of simulations included two full revolutions of the impeller (720°) (15–17).

#### Scalar Fluid Stress Analysis

High operating rotational speeds and narrow (blade tip) clearances between the rotating and stationary regions of the centrifugal pump may produce unacceptable shear stresses and result in damage to red blood cells. This blood trauma may, in turn, activate platelets and trigger clot initiation downstream, contributing to potential thromboemboli. Irregular flow patterns also contribute to the risk of hemolysis and thrombosis by increasing levels of fluid stresses. Thus, scalar stress values were numerically estimated during these simulations along with corresponding blood damage indices. An estimation of the scalar fluid stress levels in blood pumps was proposed by Bludszweit et al. (21, 22). This approach leverages comparative stress theory to produce a mathematical expression for fluid stresses based on the von Mises yield criterion of solid mechanics theory. Equation (3) was used in this study to calculate the scalar stress (σ), which includes the components of the stress tensor and represents the level of stress experienced by the blood:


(3) $$\begin{array}{c} \sigma ={\left(\frac{1}{6}\sum^{\text{​}}{\left(\sigma_{ii}-\sigma_{jj}\right)}^{2}+{\sum \sigma_{2}^{ij}}^{\text{​}}\right)}^{{}^{1/2}} \end{array}$$

We also examined fluid streamlines as indicators of numerically predicted fluid residence times. A power law relationship according to Equation (4) was utilized to relate the scalar stresses and residence time in the estimation of a blood damage index:


(4) $$\begin{array}{r} D=\overset{Outlet}{\underset{Inlet}{\sum }}1.8x10^{-6}*\sigma^{1.991}*\Delta t^{0.765} \end{array}$$

where “Inlet” and “Outlet” represent the inflow and outflow faces of the pump domain and the fluid inlet region to the bundle, D is the damage index, σ signifies the scalar stress and t is the exposure time (23). This provides a statistical estimation of the probability for blood damage to occur from the inlet to the outlet of the centrifugal pump. Using this power law relationship between the scalar stress level and the exposure time, a blood damage index was estimated (23). We assess a maximum scalar stress value of 425 Pa and a maximum residence time of 600 ms as our design threshold (24, 25). In this analysis, we track the stress history of the fluid *via* particle streamlines. The accumulation of stress and exposure time was discretely summed along the streamlines to evaluate the potential for blood damage. This approach provides a statistical estimate of damage to blood cells traveling through this blood pump using a power law equation, as described in (13, 14).

### Prototype Manufacturing

**Figure 5:**
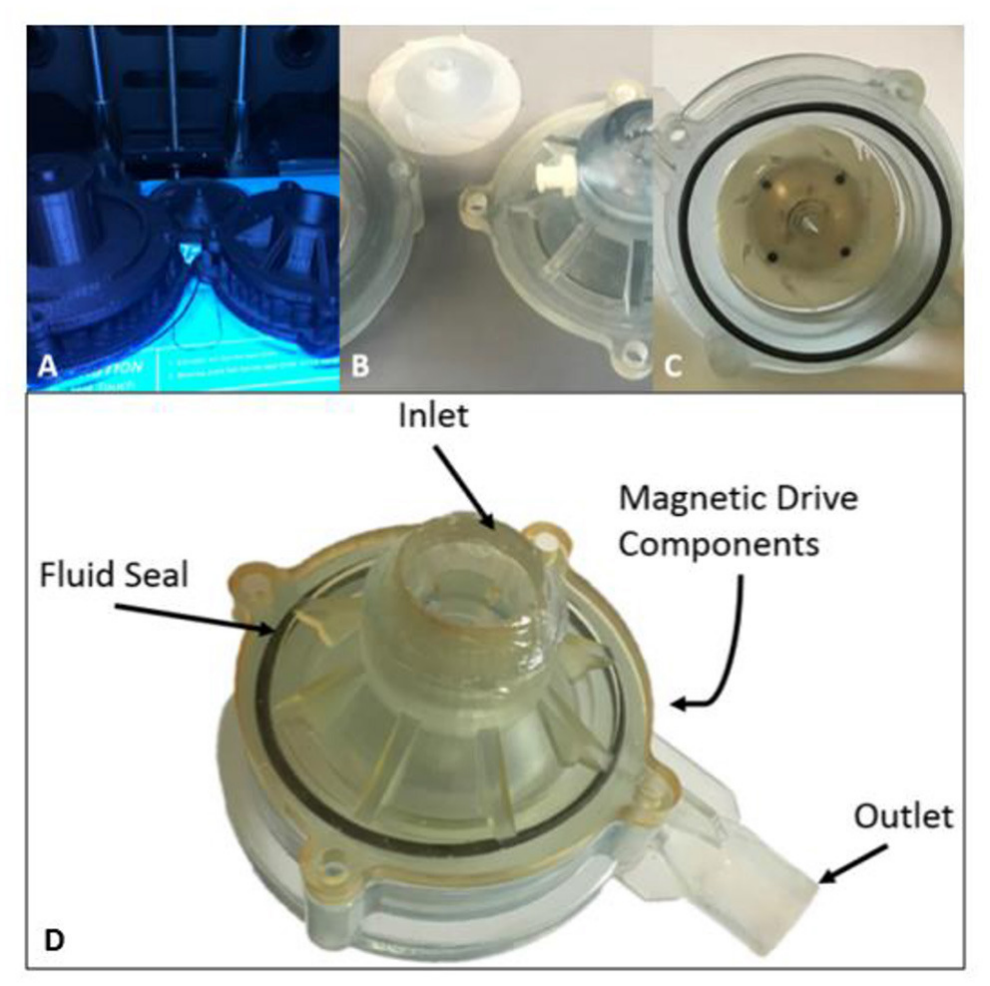
Design Progression of Magnetically Levitated Centrifugal Pump Prototypes. **(A)** Initial polylactic acid (PLA) extruded pro-totypes, **(B)** Stereolithographic 3D printing prototypes machined for testing, **(C)** Final pump prototype with point and support stabilization, **(D)** Top inlet volute with fluid seal in place and magnetic drive components to support impeller.

After modeling, the centrifugal impeller and volutes were prototyped from watershed resin material using stereolithographic techniques (Figure 5). The centrifugal impeller, made by rapid prototyping, was attached to a set of diametrically polarized magnets, designed and manufactured by our team. The lower housing of the centrifugal pump, containing both the outlet and outlet volute, was adapted to encapsulate the magnets inside of the pump housing. The prototypes were then fitted with fluid seals and tested in existing hydraulic and hemolytic test loops to characterize the pressure-flow pump performance and hemolytic potential.

### Hydraulic Testing

**Figure 6:**
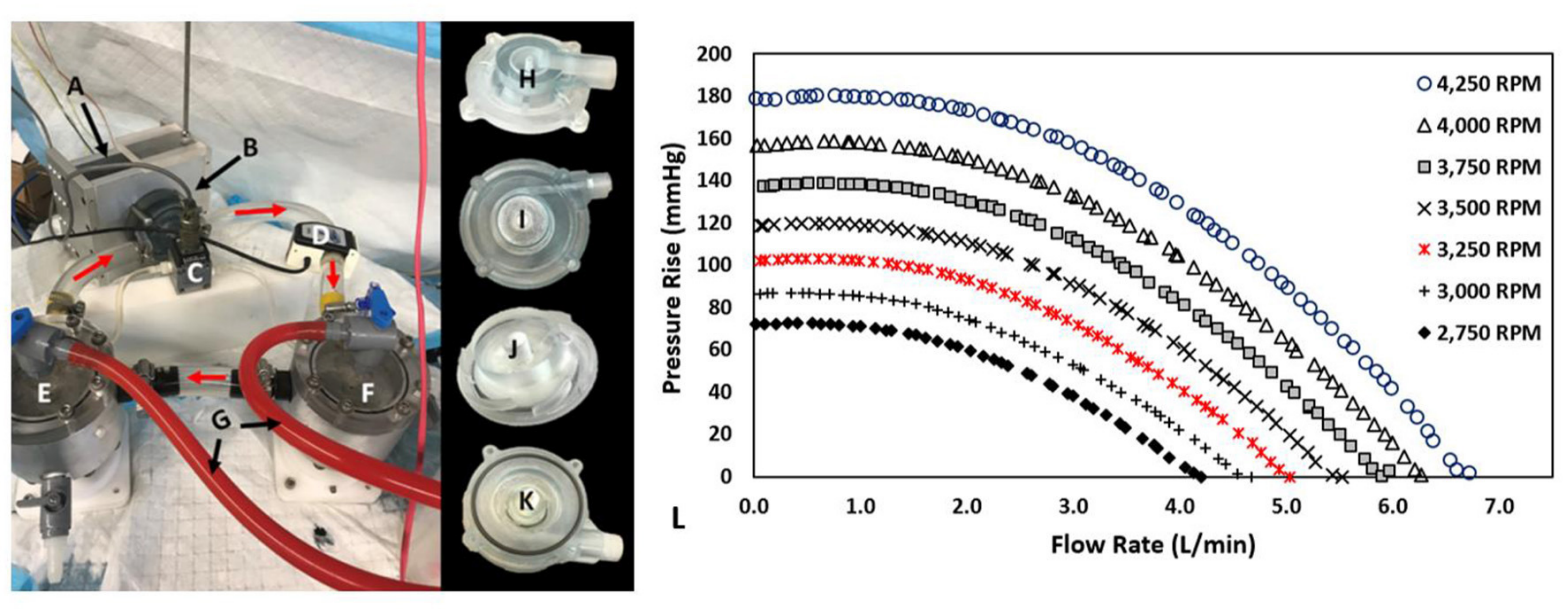
Hydraulic Test Rig, Centrifugal Prototype, and Performance Results: **(A)** Magnetically Levitated Drive System, **(B)** centrifugal blood pump, **(C)** Validyne differential pressure transducer, **(D)** Transonic Ultrasonic flow probe, **(E)** Inlet tank, **(F)** Outlet tank, **(G)** Air bleed hoses, **(H)** Inlet volute, **(I)** Outlet volute, **(J)** Impeller, **(K)** Partially assembled prototype, **(L)** Prototype Performance Results: 0-6.7 L/min at 0.5-180.3 mmHg for 2,750-4,250 RPM.

The centrifugal prototype was hydraulically evaluated utilizing a blood analog solution composed of water and glycerol (standard 60/40% v/v). Figures 6A–K illustrates the test rig configuration and pump prototype. This analog fluid was calibrated to have a measured viscosity and density of ~0.00353 Pa^*^s and 1,054 kg/m^3^, respectively. After filling the hydraulic test-rig, we removed entrained air bubbles. The test loop consisted of two reservoir tanks (inlet and outlet tank) with the pump located between them and a tubing clamp to adjust resistance and therefore flow in the circuit. Each tank was connected to a differential pressure transducer (Validyne, Northridge, CA, USA) to measure the pressure rise across the pump prototypes. Flow was measured using an external flow sensor (Transonic, Ithaca, NY, USA). A BLDC motor and controller (Faulhaber GmbH, Schonaich, Germany) was used in conjunction with the integrated magnets to induce impeller rotation (13). We collected flow rates and pressure measurements at rotational speeds from 2,750 to 4,250 RPM in 250 RPM intervals. Data were collected using a data acquisition (DAQ) board (LabJack Corp., Lakewood, CO, USA).

### Hemolytic Experiments

**Figure 7:**
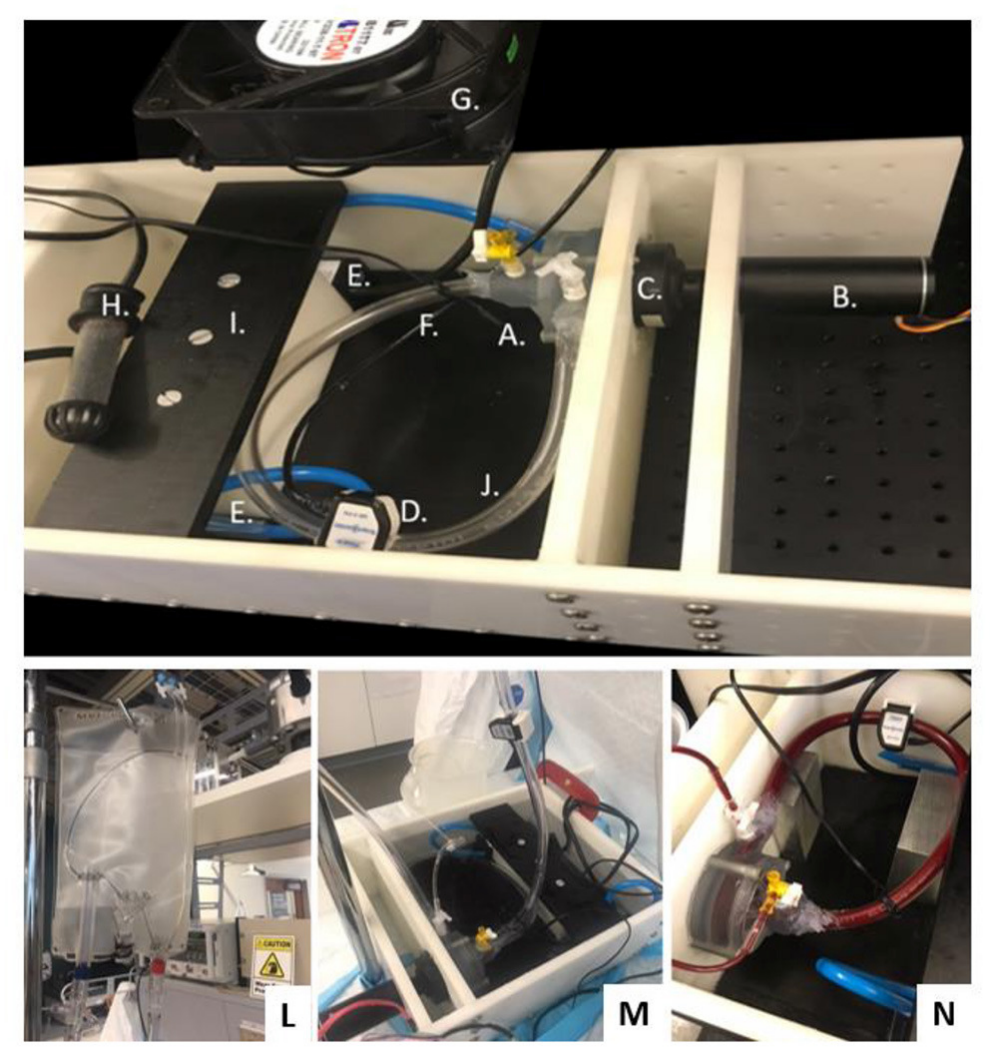
Magnetically Levitated Centrifugal Pump for Hemolysis Testing: **(A)** Centrifugal Pump (Inlet Volute, Outlet Volute, Fluid Seal, Magnetic Drive Impeller and Ceramic Thrust Bearings), **(B)** Brushless DC Motor, **(C)** No Contact Diametrically Magnetized Cup Magnet, **(D)** Flow Sensor, **(E)** Heat Bath Circulation Pumps, **(F)** Waterproof Thermocouple, **(G)** Cooling Fan, **(H)** Heater, **(I)** Heath Bath Partition, **(J)** Heparin Coated Tubing, **(K)** Leur-Lok Fill and Draw Ports, **(L)** Commercial blood bag as supply reservoir, **(M)** Connected set-up, **(N)** Pump placement and filled test rig.

Figure 7 illustrates the experimental configuration for the *in vitro* hemolysis testing. The experiments were completed using bovine blood, selected in accordance with ASTM 1830-97 (Standard Practice for Selection of Blood for *in vitro* Evaluation of Blood Pumps). The accumulated levels of plasma free hemoglobin (pfHb) were measured hourly for 4-h using the optical Cripps method (26) to characterize the degree of hemolysis for the centrifugal prototype. Experiments (*n* = 6) were performed according to our lab protocol, which follows ASTM 1841-97 (Standard Practice for Assessment of Hemolysis in Continuous Flow Blood Pumps). The flow loop was partially submerged in a 37°C water bath, controlled with a thermocouple and heating element, to maintain the temperature of the blood at or near 37°C for the duration of the experiment. Prior to filling, the flow loop was filled with phosphate buffered saline (PBS) solution to wet the internal surfaces of the loop. The bovine blood (Lampire Laboratories, Pipersville, PA, USA) was collected *via* venipuncture and shipped in a standard 1,000 *mL* bags containing citrate phosphate dextrose adenine (CDPA) anticoagulant. The blood bags were slowly warmed to 37°C in a water bath prior to the experiment.

After quickly draining the PBS solution, blood was introduced into the loop through a 150-micron filter. Once full, a blood sample was collected for the baseline measurement (initial condition, time = 0). The pump was started and slowly brought to operational rotational speed over 15 min, yielding a flow of about 2 L/min. At each 30-min interval, ~2–4 *mL* of blood aliquot was collected from the inlet tank. Two capillary tubes were filled using this sample and then placed into a centrifuge (Hettich Haematokrit 210 centrifuge, Oxford, CT). After rotation (8 min at 8,000 RPM), visual inspection using the 210 centrifuge tool itself enabled us to measure the hematocrit. Measurements were taken twice in parallel and averaged.

The remaining blood was then transferred to Covidien EDTA blood collection tubes and centrifuged for 8 min at 4,000 RPM to separate the plasma layer from the packed red cells. Using a transfer pipette, the plasma layer was collected for spectrophotometric analysis. The optical density or absorbance was measured at 576.5 *nm*, 560 *nm*, and 593 *nm*, using a Genesys 10Vis spectrophotometer (ThermoFisher Scientific, Waltham, MA). We determined the pfHb based on the weighted difference in absorbance measurements, according to the Cripps method in Equation (4) (26):


(5) $$\begin{array}{r} pfHb_{}\left(mg/dL\right)=177.6_{}\times \left[A_{1}-\left(A_{2}+A_{3}\right)/2\right] \end{array}$$

where A_1_ corresponds to the absorbance at wavelength (λ_1_) of 576.5 *nm*, A_2_ reflects the absorbance at wavelength (λ_2_) of 560.0 *nm*, and A_3_ is the absorbance wavelength (λ_3_) of 593.0 *nm*. The normalized index of hemolysis (N.I.H) was calculated using the following standard relationship in Equation (5):


(6) $$\begin{array}{r} N.I.H\left(g/100L\right)=V*\Delta pfHb*\frac{\left(100-Hct\right)}{100}*\frac{100}{\Delta t*Q} \end{array}$$

where V*Δ*pfHb* signifies the increase in plasma free hemoglobin (*g/L*) over the sampling time interval, Hct is the blood hematocrit (%), Δt corresponds to the sampling time in minutes, Q represents the flow rate in liters per minute, and *V* is the circulating volume of the hydraulic loop in liters.

### Data Analysis

Simulation results were assessed qualitatively and quantitively. We estimated the pressure generation, scalar stress levels, and fluid forces exerted on the magnetically levitated impeller. For the transient runs, data analysis was performed on the second revolution results for each case. To quantitatively compare the pressure-flow performance of the models with the prototype performance, we used a regression analysis that is detailed in (27). This approach involves the calculation of non-dimensional pressure and flow coefficients for the data sets. The regression model includes characteristic constants (β_0_, β_1_, β_2_), as described in this equation:


(7) $$\begin{array}{r} \psi_{TYPE}=\beta_{2}\left({\varphi_{TYPE}}^{2}\right)+\beta_{1}\left(\varphi_{TYPE}\right)+\beta_{0} \end{array}$$

The subscript “Type” is generic since comparisons were made across data sets of computational predictions and experiments. The F-test for the regression analysis was used to assess the significance of the coefficients and polynomial models based on the data normality. We then determined an average and maximum deviation between data sets.

The hemolytic data were analyzed according to standard ANOVA principles using SPSS. A Shapiro-Wilk normality test was completed in conjunction with a Tukey-Hoaglin outlier test. A test of homogeneity of variance was conducted to analyze the variance between the six different experimental results for N.I.H. values, and heteroskedasticity test was utilized to assess significance of time point values across experiments. All statistical analyses were based on a preset α value of 0.05.

## Computational Results

### Mesh Quality

The mesh for all fluid domains was created using the mesh generator within ANSYS, and the required mesh density was determined using a grid independence study to determine when the grid resolution was no longer affected the physics being modeled (<3% variable variance). All mesh elements were required to have an aspect ratio <100, Jacobian ratio <40, a skewness <0.25 and an element quality measure >0.75; these were achieved for all model constructs. The k-epsilon model satisfied the y+ mesh requirement of being >11 along all walls and surfaces. Inflation layers were also used to achieve the SST turbulence model requirement (y+ <2). This mesh-build analysis indicated that ~3 million elements was sufficient to achieve grid independence.

### Steady Flow Simulation Findings

Over 400 simulations were performed on the centrifugal pump using the model in Figure 4A. Figures 4B,C illustrate the pressure-flow performance for this pump design over a range of flow rates and rotational speeds for both the SST and k-epsilon turbulence models. Each data point corresponds to a steady state simulation for a specific flow rate and rotational speed. The pressure rise across the centrifugal pump design was determined for flow rates of 0.5 to 6 L/min for 2,750 to 4,250 RPM. A pressure rise of 10 to 145 *mmHg* was achieved over these operating conditions (Figure 4B). The design point of 3 L/min at 3,500 RPM demonstrated a pressure rise of ~95 mmHg. Pressure rise was found to decrease with increasing flow rate and was higher at faster rotational speeds. This trend, as expected, was observed for both turbulence models. The pressure performance curves demonstrate the pump's ability to deliver an adequate flow range with target pressure rises at reasonable rotational speeds. For flow rates <3 L/min, the calculated expected pressure rises for the two turbulence models were observed to be 12–23% *mmHg* different in magnitude. At flow rates >3 L/min, the pressure rises were within 4–8% of each other, as seen in Figure 4C.

Axial fluid forces exerted on the magnetically levitated impeller rotor surface ranged from 0.4 to 2.5 N, and the radial fluid forces were found to be much lower in magnitude, ranging from 0.17 N to 0.84 N. As expected, higher axial fluid forces were observed for operating conditions yielding higher pressure generations. Hydraulic efficiencies (10–28%) were determined using the computational torque, enabling an estimation of the mechanical power usage, which ranged from 0.79 Watts to 4.58 Watts.

**Figure 8:**
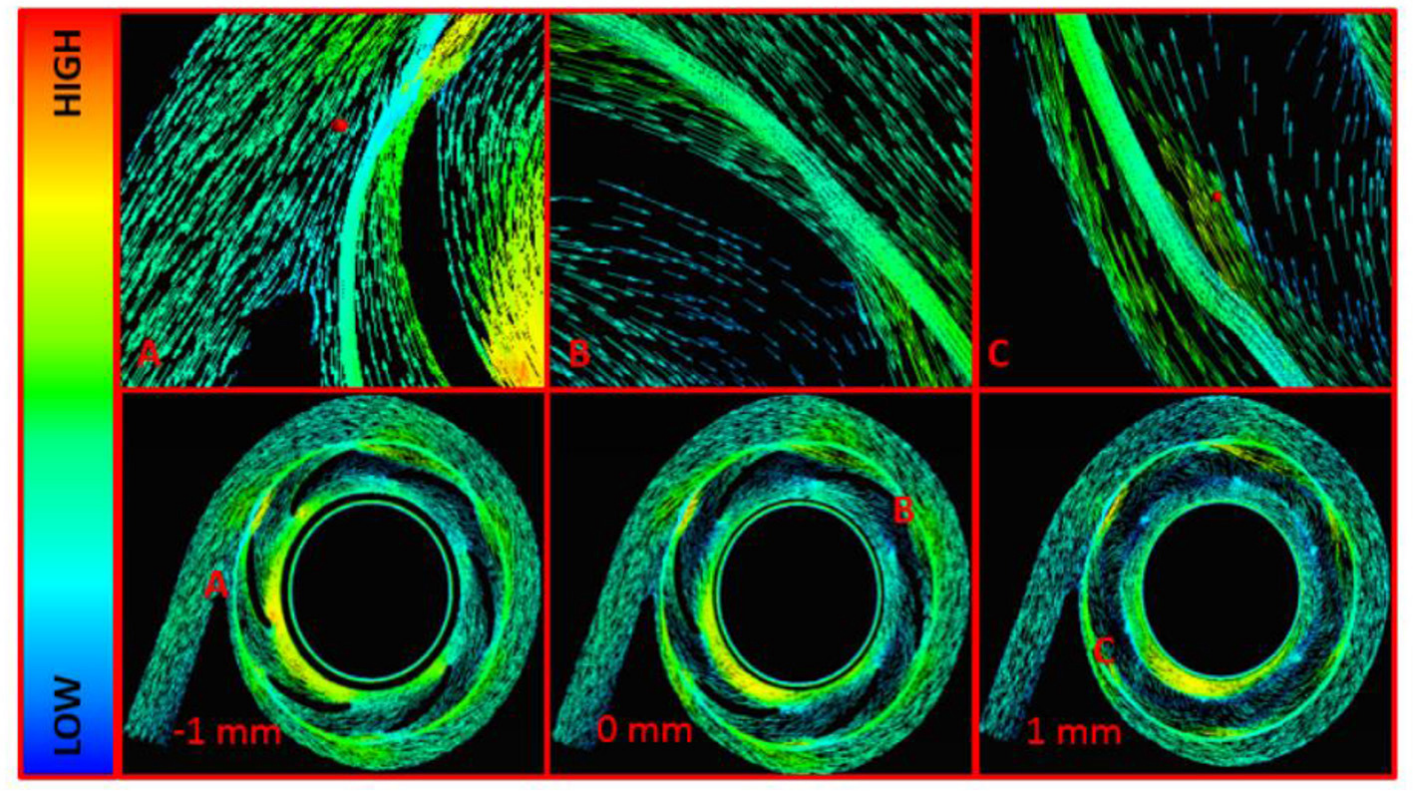
Velocity Vectors for the Centrifugal Pump Model at 3,500 RPM and 3 L/min. **(A)** Cutwater Region, **(B)** Impeller Blade Tip, **(C)** Secondary Flow Path and Impeller Cutwater Region at the Outlet Volute. These are midspan views across the centrifugal blood pump. The central midspan is indicated at 0 mm, and two additional midspan views are shown 1 mm above and -1 mm below the central midspan.

Fluid velocities were inspected in all models and at all operating conditions. Figure 8 displays the fluid velocity vectors for the centrifugal pump model at the design point. Highlighted regions include the cutwater at the outlet of the pump, impeller tip clearance, and secondary flow path. These regions demonstrated irregular, vortical flow conditions, per the computational predictions. Outside of these regions, blood flow transitioned well from region to region across the centrifugal blood pump.

Tables 1, 2 indicate the scalar stress estimations and blood damage index calculations. All operating conditions resulted in blood damage indices of <2%, the threshold level for our designs. Approximately 99% of the computational walls and surfaces had scalar stresses below 425 Pascals. The fluid residence times, measured *via* streamlines, were all <600 ms, meeting the target requirement. As expected, higher rotational speeds yielded higher scalar stress levels. Higher scalar stresses were found in the blade tip clearances, at the leading edge of the blades, near the cutwater region in the outlet volute, and on the suction side (i.e., backside) of the impeller blades.

**Table 1:** Fluid stress level and blood damage index estimations for the centrifugal pump model.

**Flow rate**	**Scalar stress (Pa)**		**Damage index (%)**	
**(L/min)**	**Avg**	**% > 425**	**Mean**	**Max**
**Operating rotational speed of 4,250 RPM**				
3	59.9	0.7	0.170	1.12
4	66.6	1.0	0.193	1.06
5	66.5	1.0	0.178	0.80
**Flow rate**	**Scalar stress (Pa)**		**Damage index (%)**	
**(L/min)**	**Avg**	**%** **> 425**	**Mean**	**Max**
**Operating rotational speed of 3,500 RPM**				
3	42.0	0.08	0.083	0.66
4	50.2	0.27	0.099	0.53
5	54.0	0.55	0.098	0.58

*Flow rates of 3–5 L/min at Rotational Speeds of 3,500 RPM and 4,250 RPM*.

**Table 2:** Fluid stress level and blood damage index estimations for the centrifugal pump model.

	**Scalar stress (Pa)**		**Damage index (%)**	
**RPM**	**Avg**	**% > 425**	**Mean**	**Max**
**Operating flow rate of 3 L/min**				
4,250	59.9	0.7	0.170	1.12
4,000	53.6	0.4	0.141	0.81
3,750	47.7	0.2	0.129	0.15
3,500	42.0	0.1	0.083	0.66

*Rotational Speeds of 3,500–4,250 RPM at 3 L/min*.

### Transient Flow Simulation Findings

#### Quasi-Steady Study on the Centrifugal Impeller

Figure 4E illustrates the computational predictions for the quasi-steady analysis of the centrifugal blood pump and the impact on pressure generation as a function of rotational position of the impeller blades. Sixty simulations were completed for rotational increments of 6°. This study was performed at 3,500 RPM and 3 *L/min*. Six peak pressure generations were observed, and these peak pressure generations occurred at every 60°. The peaks correlate with blade number, and a shift in the pressure rise was observed from 86 to 95 *mmHg*, indicating a blade passage influence in the pressure rise across the pump.

#### TRSI Results

Figure 4F demonstrates the TRSI simulations for a rotational speed of 3,500 RPM at 3 *L/min*. The average pressure generation was found to range from 74 to 96 *mmHg*, which is a much wider range than indicated by the quasi-steady study. Pressure fluctuations due to the blade passage frequency are more significant and can be observed with the expected 6 peaks in a full rotation. The axial and radial fluid forces ranged from 1.71 to 1.93 N, and 0.16 to 0.79 N, respectively.

### Hydraulic Results

Figure 6L displays the pressure-flow hydraulic performance results for the testing speeds of 2,750–4,250 RPM in 250 RPM intervals. The magnetically levitated centrifugal prototype delivered 0–6.75 L/min at 0–182 mmHg over those rotational speeds. Higher rotational speeds led to more substantial pressure rises at a given rotational speed. Moreover, higher flow rates produced lower pressure rises at a given rotational speed. These trends met expectations for centrifugal pumps. The full flow range of testing for 2 rotational speeds were re-measured, and the pressure-flow performance data demonstrated repeatability.

### Hemolytic Results

Over each 4-h hemolytic test period, the rotational speed, temperature and flow rate were held constant, and we drew test samples every 30 min. The bovine blood in the circuit was maintained at a temperature of 36.31 ± 1.94°C, a flow rate of 2.06 ± 0.06 L/min and rotational speed of 1,080 ± 228 RPM. The initial bovine blood hematocrit values ranged between 30 and 38% and slightly decrease over the duration of the study; an average hematocrit drop of 1.8% ± 0.7% (*p* < 0.5) was measured for all experiments. The pfHb levels increased linearly over the experiment 4-hr duration, as expected. The Shapiro–Wilk test supported a normality assumption (*p* > 0.05), and the pfHb concentration linearity was verified *via* regression statistics using F-test and coefficient *t*-test (*p* < 0.5). The significance and independence of each hourly average pfHb level was also assessed. No outliers were determined per the Tukey-Hoaglin outlier test. A test of homogeneity of variance was showed that there was equal variance between experimental data sets (*p* > 0.5). An F-test for heteroskedasticity demonstrated that the variance of the errors does not depend on time, thus further supporting the significance and independence of each hourly average pfHb measurement. Table 3 shows the N.I.H determinations for each experiment, which ranged from 0.06 g/100 L to 0.12 g/100L. The average N.I.H value (*n* = 6) for all of the experiments was determined to be 0.09 ± 0.02 g/100 L (*p* < 0.05).

**Table 3:** Normalized Index of Hemolysis (N.I.H) for bovine blood studies.

**Experiment**	**N.I.H (g/100 L)**
*N* = 1	0.10
*N* = 2	0.10
*N* = 3	0.12
*N* = 4	0.11
*N* = 5	0.07
*N* = 6	0.06
Ave	0.09 ± 0.02*

Repeated (n = 6) hemolytic studies at ~2 L/min using magnetically levitated centrifugal pump prototype. *p < 0.05.

## Discussion

To provide a new therapeutic solution for pediatric patients with heart failure, we are developing a new blood pump technology in the form of a continuous-flow, magnetically levitated TAH (13, 14). The overall target size of this TAH is 50 mm in diameter by 50 mm in height and has only 2 moving parts, an axial and centrifugal impeller, each levitated by magnetic suspension. Thus, this blood pump technology is expected to be able to provide extracorporeal MCS to patients < 0.65 m^2^ (i.e., <12 kg or younger than ~2 years old) and intracorporeal MCS to patients >0.65 m^2^ (i.e., >15 kg or older than ~2 years old). The axial pump mechanically supports the pulmonary circulation, and the centrifugal impeller supports the systemic circulation. The device utilizes the latest generation of magnetic bearing technology to levitate the impellers, enabling a longer operational lifespan and wider clearances between the rotating impeller surfaces and pump housing (about 5 × wider than mechanical bearings). These wider clearances reduce fluid shear stresses and lower the risk of thrombosis and hemolysis. This design also avoids the use of the mechanical or biologic valves that can fail prematurely.

Initial designs have been developed for both the axial and centrifugal pumps (13, 14). Since the axial region and centrifugal fluid domains are separate and independent from each other, the pumps can initially be developed separately. The data presented in this paper, consisting of both computational and experimental studies, has concentrated on the design and development of the centrifugal pump and presents the latest developmental progress.

In the computational studies, two turbulence models were employed: k-epsilon and SST models. A quantitative comparison of the computational predictions, using both turbulence models, and the experimental measurements of the centrifugal pump prototype was performed. Figure 4D illustrates this comparison. For flow rates <3 *L/min*, the SST model correlated to within 10% of the experimental measurements, and the k-epsilon model correlated to within 19% of the experimental measurements. The SST and k-epsilon models correlated to within 5–8% of the experimental measurements for flow rates >3 *L/min*. The SST turbulence model utilizes a blending function to transition between near-wall modeling equations and bulk k-epsilon modeling equations. It is expected that as the flow rate increases, yielding higher Reynolds numbers, the SST and k-epsilon models would predict more similar results. This was observed computationally. Deviations between CFD and experiments are expected. While the choice of turbulence model is important to the accuracy of the simulations, there is no definitive approach to determine which turbulence model is optimally suited for miniature blood pumps; where the blood flow conditions are highly dynamic and likely fluctuate among laminar, unsteady transitional, and truly turbulent flow conditions.

The only rotating component in the centrifugal pump is the impeller and its 6-bladed impeller that causes the observed blade passage frequency in the fluid physics. This effect was observed using both quasi-steady analysis and the transient TRSI approach. Six peak pressure rises, corresponding to the six blades, were observed in the computational data sets. Pressure fluctuations due to the blade passage frequency were more pronounced for the TRSI simulations, and axial and radial fluid force ranges were wider for the TRSI study. Nevertheless, significant pressure and fluid force deviations between the quasi-steady and TRSI simulations were not found, in accordance with prior studies (16).

Per the hydraulic studies, the centrifugal pump prototype delivered target pressure rises and capacities to support pediatric patients. We chose the tightest tolerancing for prototype manufacturing (Applied Rapid Technologies, Fredericksburg, Virginia, USA) in support of the impeller geometry, blade tip clearance and secondary flow path dimensions. Trends among performance of the computational models and prototypes were similar for the centrifugal model and prototype testing; both data sets followed expected trends according to pump design theory (28). Computations predicted the axial fluid forces to be <3 N and radial forces of <1 N for the centrifugal pumps; these forces correlate well with levels of other magnetically-levitated centrifugal blood pumps. The higher scalar stress along the blade tips, leading edge of the impeller blades, and at the cutwater region is also to be expected due to the presence of transitional velocity gradients and proximity of rotating-stationary surfaces. After completing this study, we explored improving the mesh element in these regions and found that the scalar stress is highly sensitive to the volume skew of the elements in these regions. A recent publication also indicates shear stresses being sensitive to mesh characteristics (29). The majority of scalar stress values were below 425 Pascals, and blood damage indices were below the design criteria of 2% for all operating conditions.

The magnetically levitated centrifugal prototype incorporated touchdown surfaces on the center spindle region and a constraining secondary flow path. Thus, the N.I.H. values for the repeated 4 h studies were found to be higher than desired. Clinically approved blood pumps have demonstrated N.I.H values of <0.04 g/100 L, and our results average N.I.H was 0.09 ± 0.02 g/100 L. The hemolytic potential of our current centrifugal prototype design is problematic, and the hemolytic potential must be reduced with further design improvement. Next phase development will integrate the axial flow blood pump, and thus the spindle region and secondary flow path will be modified to address the complex fluid dynamics in these regions.

### Study Limitations and Future Work

This study has limitations that must be addressed in future work. The focus of this work has been on the centrifugal pump design, and we must improve the axial pump in future development phases. The axial blood pump is currently under redesign to reduce size, lower fluid forces on the levitated impeller, and improve energy transfer. We are in the process of combining the centrifugal prototype with the axial pump to conduct an integrated configuration for hydraulic and hemolytic testing. We must evaluate the integrated, full pediatric TAH design both computationally and experimentally to evaluate the hydraulic performance, hemolytic levels, and magnetic flux directions to assess and address any interference between suspension systems. Next phase hemolytic studies must include more flow rates and rotational speeds and be conducted for a minimum of 6 h study durations. Inflow and outflow cannulae are currently being designed using 3-D MRI images of human thoracic cavities. Moreover, we have constructed a lumped parameter model of the cardiovascular system such that we can include downstream and upstream boundary conditions in a multi-scale computational platform. The balance of blood flow and preload distribution between the pulmonary and systemic circulations is also an ongoing challenge for continuous flow TAHs, especially for those that utilize a single-rotor configuration. In this configuration, the axial and centrifugal pumps are separate and thus can be controlled independently; nevertheless, we must determine optimal control algorithms during the next development phase. Fit and placement will also be virtually examined, and control algorithms will be devised to ensure either RPM for pulsatility in the outflow for long-term mechanical circulatory assistance or to maintain continuous flow during acute support scenarios. A focus is also currently on the motor and magnetic suspension system integration and a size reduction in preparation for acute animal experiments during next steps.

## Conclusions

Clinically-available blood pumps and total artificial hearts for pediatric patients continue to lag behind those developed for adults. To address this unmet clinical need, we are developing a hybrid-design, continuous-flow, magnetically levitated, pediatric total artificial heart (TAH). The hybrid TAH design integrates both an axial and centrifugal blood pump within a single, compact housing. The centrifugal pump rotates around the separate axial pump domain, and both impellers rotate around a common central axis. Here, we performed computational studies and prototyping testing for the centrifugal pump component. The computational studies and the prototype testing demonstrated that the centrifugal design is effective; design requirements for the centrifugal pump were achieved. The pressure-flow performance, axial and radial fluid forces, scalar stresses, blood damage indices and mechanical power consumption estimates fell within target design requirements, except that the hemolytic potential must be reduced with further design improvement. These data support the next phase of development for the motor and magnetic suspension of the centrifugal pump and integration of the axial flow blood pump component.

## Data Availability Statement

The raw data supporting the conclusions of this article will be made available by the authors, without undue reservation.

## Author Contributions

AT and CF: concept and design, data collection, data analysis and interpretation, critical revision of article, and approval of article. TP: data collection, data analysis and interpretation, drafting article, critical revision of article, and approval of article. MH: data collection, data analysis and interpretation, drafting article, critical revision of article, and approval of article. RS, JR, SD, and VT: data analysis and interpretation, critical revision of article, and approval of article. All authors contributed to the article and approved the submitted version.

## Conflict of Interest

The authors declare to disclose that the lead and corresponding authors (CF and AT) from the BioCirc Research Laboratory are inventors on patents for the Drexel Dragon Heart blood pumps (two awarded USPTO patents and patent applications that are currently under consideration by the USPTO). JR discloses that he has served as a consultant for Abiomed, MyoKardia, Merck, Bayer, and Novartis. The remaining authors declare that the research was conducted in the absence of any commercial or financial relationships that could be construed as a potential conflict of interest.

## Publisher's Note

All claims expressed in this article are solely those of the authors and do not necessarily represent those of their affiliated organizations, or those of the publisher, the editors and the reviewers. Any product that may be evaluated in this article, or claim that may be made by its manufacturer, is not guaranteed or endorsed by the publisher.
